# Resistance Exercise for Improving Running Economy and Running Biomechanics and Decreasing Running-Related Injury Risk: A Narrative Review

**DOI:** 10.3390/sports10070098

**Published:** 2022-06-24

**Authors:** Anja Šuc, Pija Šarko, Jernej Pleša, Žiga Kozinc

**Affiliations:** 1 Faculty of Health Sciences, University of Primorska, Polje 42, SI-6310 Izola, Slovenia; 97210493@student.upr.si (A.Š.); 97210482@student.upr.si (P.Š.); 97200425@student.upr.si (J.P.); 2 Andrej Marušič Institute, University of Primorska, Muzejski trg 2, SI-6000 Koper, Slovenia

**Keywords:** runners, biomechanics, training, prevention, exercise

## Abstract

It is well-accepted that at least a certain amount of resistance exercise (RE) is recommended for most endurance athletes. In this review, we aim to summarize the evidence regarding the effects of RE on running economy, running biomechanics, and running-related injury risk in endurance runners. The evidence robustly shows that lower limb RE is effective for improving running economy and performance, with a combination of strength and plyometric training being recommended to improve RE. Isometric training is also emerging as a possible alternative to implement during periods of high overall training load. Lower limb RE may change some aspects of joint kinematics during running; however, the evidence regarding the effects on kinetics is limited. Lower limb RE may help reduce running-related injury risk, but further evidence is needed.

## 1. Introduction

The utility of resistance exercise (RE) for endurance athletes has been a matter of debate among sport scientists for a long time. Today, it is well-accepted that at least a certain amount of RE is recommended for most endurance athletes. In this paper, we focus on the evidence regarding the effects of RE for endurance runners. We address the effect of RE on running economy, running biomechanics, and injury risk.

Highly trained endurance runners normally have similar and very high V̇O_2_max values (70–80 mL/kg/min with relatively small variations between athletes [1,2]); therefore, their performance depends in large part on their running economy [3]. Running economy is defined as the amount of oxygen consumed (V̇O_2_) at a given submaximal running speed and is expressed in mL/kg^−1^/min^−1^ [4]. Simply put, at a given speed, a runner with a better running economy consumes less oxygen compared to a runner with a lower running economy [5]. Studies typically report a very strong correlation between running economy and endurance running performance (r = 0.8–0.9) [6]. A plethora of modifiable determinants of running economy have been reported, including stride length, stride rates, contact times, lower vertical oscillation, greater leg stiffness, lower limb kinematics, alignment of the ground reaction force and lower limb axis during the propulsive phase, arm swing, lower coactivation, and firm footwear [7,8,9]. In addition, several anthropometric characteristics such as height, limb dimensions, and body composition have been suggested to influence running economy [10]. In relation to this article, neuromuscular properties such as muscle strength, elastic energy utilization, eccentric power, and leg muscle stiffness are of primary interest [11]. The reutilization of elastic energy is a mechanism that can substantially reduce energy consumption during running [12]. The ability to reuse elastic energy during the propulsive phase of the running cycle is determined by reactive strength and leg muscle stiffness [7,13]. Reactive strength is the ability of the musculoskeletal system to produce a large force with concentric contraction immediately after rapid eccentric contraction, also known as eccentric–concentric contraction or the stretch–shortening cycle (SSC) [14].

Muscle stiffness is defined as the ability of a system to resist deformation and is calculated as the ratio of the force produced to the change in muscle length. It represents the integrative action of muscles, tendons, and ligaments that work together as a spring that stores and reuses elastic force [15]. The greater the muscle stiffness, the less muscle activation is required to transfer energy during running, thus reducing energy expenditure and improving running economy [16]. Stiffness on the muscle level is difficult to assess during dynamic tasks, such as running. Consequently, studies have typically calculated quasi-stiffness, which is defined as the ability of the human body to resist displacement [17]. For instance, vertical quasi-stiffness is defined as the ratio between changes in the ground reaction force and vertical displacement of the center of mass, while joint quasi-stiffness describes the angular displacement within a joint in relation to joint moments [18,19]. RE can increase reactive strength and quasi-stiffness (although the results on directly measured muscle stiffness are inconclusive [20]). The increase in quasi-stiffness is one of the primary candidates for underlying the improvement in running economy after RE [21], which is also corroborated by simulation studies demonstrating that a decrease in stiffness results in increased energy expenditure during running [22].

Everything else being equal, stiffness is inversely related to flexibility; thus, it is not surprising that studies have shown an inverse relationship between flexibility and running economy [23,24]. On the other hand, it seems that if muscle stiffness is preserved after stretching intervention, running economy is not affected [25]. Given that sufficient range of motion (particularly at the hip and ankle joints) is needed for optimal running technique, performing some flexibility exercises in addition to RE could be beneficial. The interested reader is referred to the review paper on the acute and chronic effects of stretching on running economy by Carter et al. [25].

The incidence of running-related injuries ranges between 3% and 85% [26]. The majority of these injuries are overuse/chronic [27], with the highest incidence reported for iliotibial band syndrome (ITBS), patellofemoral pain syndrome (PFPS), Achilles tendinitis, plantar fasciitis, and medial tibial stress syndrome [28]. According to Troy et al. [29], suboptimal biomechanics results in an uneven distribution of forces between body segments. This is seen particularly in people with PFPS and ITBS, where unevenly distributed forces increase stress on a particular part of a structure [30,31]. One study demonstrated that PFPS is associated with weak muscles that control the hip and muscles that regulate pelvic movement and external knee rotation [32]. Additionally, abnormal biomechanics has also been observed in these individuals. A similar finding was found in runners with ITBS, in whom increased internal knee rotation and increased hip displacement were observed [33].

RE seems to be a promising tool to improve running economy and potentially for strengthening the weakened muscles that cause altered biomechanics and, by extension, increased injury risk. Therefore, the purpose of this narrative review is to summarize the evidence of the effect of RE on running economy, running biomechanics, and running-related injury risk. Systematic reviews to date have generally shown that RE improves running economy [34]; however, most of the recent reviews focused only on the effects of strength training in highly trained runners [35], such as concurrent heavy weight and explosive training [36]. Existing reviews on running biomechanics and injury-risk typically focus on multiple interventions or interventions other than RE [37,38,39,40]. The purpose of this paper is to summarize, in a narrative manner, the available evidence on effects of different RE modalities for different domains of application related to running. We believe that this paper will outline specific gaps in the literature and also help coaches and clinicians who work with runners to design RE programs for specific purposes and goals. A search of the literature was performed in January 2022. The following keywords were used in various combinations in PubMed and Scopus databases: running, runners, running-related, injury, biomechanics, performance, and economy. Upon retrieval of the relevant full texts, potentially useful references were retrieved from the reference lists of the included articles. Both reviews and original research were considered. Studies were included if they analyzed runners, triathletes, or military personnel. Studies were excluded if the article was written in a language other than English. Although we primarily searched for randomized controlled trials and other experimental study types (i.e., assessing the effects of RE on running economy, running biomechanics, and injury-risk), we also included cross-sectional studies into the narrative where appropriate.

## 2. Resistance Exercise for Improving Running Economy and Performance

Running performance on middle and long distances depends on anthropometric, biomechanical, and physiological characteristics [41]. In his endurance performance model, Joyner considered three key physiological parameters—V̇O_2_max, blood lactate concentration, and running economy [42]. Paavolainen et al. suggested that among well-trained distance runners, running economy is a better predictor of performance than V̇O_2_max [43]. A recent systematic review showed improvements of running economy without changes in V̇O_2_max and blood lactate concentration in pre-trained athletes after RE intervention [41]. However, the results between studies differed considerably, which can be attributed to different running speeds being used for running economy assessments and other inconsistencies in the measurement protocols [41]. Di Prampero et al. demonstrated that runners improved running economy more while running at competitive speed [44]. After 8 weeks of concurrent explosive strength training and endurance training, young runners improved their anaerobic and neuromuscular properties without reducing their aerobic capacity [45]. An older systematic review supports these findings with improved running economy (+8%) in elite long-distance runners after explosive strength training. However, in untrained runners, that was not the case, as they improved only in V̇O_2_max [46]. In competitive runners, even a small improvement in running economy can have a major impact on long-distance running performance, especially in marathons and ultramarathons [46].

Cross-sectional evidence shows that muscle strength and quasi-stiffness are positively related to running economy, which supports the use of RE to improve running economy. Li et al. [47] studied the relationship between the neuromuscular properties of the lower extremities and running economy at three different speeds (12, 14, and 16 km/h). Running economy at 12, 14, and 16 km/h was significantly affected by eccentric strength in leg press (r = 0.52–0.63), as well as reactive strength (r = 0.41–0.57) and leg quasi-stiffness (r = 0.68–0.76), determined from drop jump testing. During eccentric contractions, the muscle–tendon unit is stretched, absorbing mechanical energy, which is then reused during concentric contractions [48]. As mentioned before, the action of stretching, which is immediately followed by muscle contraction, has been termed as the stretch–shortening cycle (SSC). During the SSC, the muscle works as a spring and is able to produce more force in the concentric action (compared to concentric movement alone), which is crucial for running economy [48]. Up to a 50% increase in force can occur due to energy reuse during SSC actions [49]. These research findings describe potential underlying mechanisms of improvement in running economy and performance after RE in endurance runners. In the following sections, the effects of specific RE modalities are discussed.

Although some forms of RE may elicit hypertrophic effects and consequently lead to an increase in body mass, such an effect has not been shown in most studies conducted on runners. Docherty & Sporer [50] described the concept of the interference phenomenon between concurrent endurance training and RE. Based on their review of the literature, simultaneously training for strength and endurance compromises the development of muscle strength (as compared to using only RE). However, the development of endurance (specifically aerobic power) appears to be unaffected by concurrent RE, which supports its use for endurance athletes. The fact that the body mass is unchanged in most RE intervention studies is important to point out, as the effect of RE could also be accompanied by an increased muscle mass and thus increased total body mass, which could negatively affect the relative physiological determinants of endurance, such as running economy and V̇O_2_max [51]. Li et al. [11] studied the impact of complex training, which included maximum strength training and plyometric training, on running economy and came to the same conclusion. Namely, body composition did not change with training, which indicated that the adjustment to resistance training was mainly due to adaptations on the level of the nervous system (e.g., increased frequency of motor unit firing and increased recruitment of motor units) [52].

### 2.1. Strength Training

Five studies included in a recent systematic review [35] reported a positive effect of strength training on running economy in highly trained endurance runners. The duration of the interventions ranged from 8 to 12 weeks, with one study using high loads (85% 1RM) and the other four using moderate loads (40–70% 1RM). The addition of RE to the endurance training of well-trained endurance runners improved running economy (+4%) after 10 weeks without changes in body mass and V̇O_2_ [53]. In addition, an 8-week intervention of heavy weight training on well-trained endurance runners improved running economy (+5%) and postponed the time to exhaustion, while no changes in body mass were reported [54]. A study by Beattie et al. [51] performed on endurance runners also found that RE performed in addition to endurance training improved running economy and V̇O_2_max without significant changes in body composition, which was also replicated in other research [41,55]. A recent study [55] found similar results in triathletes, who improved their maximal strength, cycling economy, and running economy in a 26-week concurrent endurance and RE program. Additionally, Alcaraz-Ibañez and Rodríguez-Pérez [41] found no signs of overtraining when RE was added onto the training schedule of previously trained endurance athletes. However, there were inconsistencies in improvements among athletes, which might be a consequence of different characteristics of the applied RE.

### 2.2. Plyometric Training

Several studies have also investigated the effects of plyometric training on running economy. A 9-week intervention of plyometric training in highly trained distance runners improved running economy at 18 km/h^−1^ (+4.1%; *p* = 0.02), accompanied by trends for improved 5-jump plyometric test performance (+14.7%; *p* = 0.11) and a lower VO_2_max–speed slope (14%; *p* = 0.120) [56]. A 6-week plyometric training intervention improved running economy, CMJ height, 5-jump plyometric test performance, and lower leg quasi-stiffness [57]. Importantly, the increase in quasi-stiffness was correlated with improvements in running economy. Increasing lower leg quasi-stiffness and reactive strength through plyometric training enables better utilization of the SSC [11,57]. Interestingly, Turner et al. [58] reported that moderately trained endurance runners improved running economy after 6 weeks of plyometric training; however, there was no improvement in vertical jump performance. This could have been due to the shorter duration of the plyometric training (10–15 min), which was not primarily focused on improving vertical jumping performance. In a systematic review of five articles [59], it was reported that both plyometric training and heavy weight RE improved running economy by ~5%. In some studies, a short-term (4-week) heavy weight training intervention improved running economy (+6.3%; 44.3 ± 4.9 to 47.3 ± 6.8 mlkg^−1^min^−1^), but plyometric training did not [60].

### 2.3. Combination of Different RE Modialities

Skovgaard et al. [61] studied the effect of a combination of strength training and speed endurance training on the athletic performance of runners. They concluded that this type of training improves running economy without increasing the risk of overtraining, which is consistent with other research findings [54,62]. Li et al. [11] found that 6 weeks of complex training, which combines heavy weight training and plyometric exercises with endurance training, improved maximum muscle strength and power, running economy, and velocity at VO_2_max in well-trained endurance runners, which was not the case in resistance training, which consisted of only strength endurance exercises. A study by Barnes et al. [63] examined the impact of heavy weight training and plyometric training on endurance runners during the competitive period. They found that, in accordance to previous research, the body composition did not change, but there were some unexpected results—the effects of the intervention on running economy varied significantly between male and female participants. In particular, the addition of plyometric or heavy weight training during the competition period had a negative impact on the competitive performance in males, while a positive effect was seen in female endurance runners [63]. Further research is urgently needed to reveal possible differences in adaptations to RE between male and female runners.

### 2.4. Isometric Training

It has been suggested that isometric training could elicit similar or even higher adaptations in running economy than plyometric training. Two older studies reported an improvement in running economy (~4–7%) after isometric plantar flexor strength training [64,65]. Recently, Lum et al. [66] directly compared the effects of isometric and plyometric training in a 6-week interventional study. Although both training types improved running economy, the isometric group surprisingly improved more, which was also reflected in a higher maximal aerobic speed. It could be that isometric training is superior in terms of increasing muscle/tendon stiffness, which was indicated in some of the previous studies [67]; however, leg quasi-stiffness during running in the study by Lum et al. [66] was not changed in either group. While further research is clearly needed, the authors concluded that isometric training could be a good alternative to plyometric training, especially during training cycles when runners already experience a high volume of load during their regular endurance training.

### 2.5. RE Coupled with Vibration Training

Another attractive avenue for research is the inclusion of vibration platforms into the training regimen. Vibration exposure is thought to affect the central and peripheral nerves and enhance neuromuscular activity, thereby increasing force production [68]. Among other things, the improvement of running economy is thought to be due to altered neuromuscular properties leading to increased muscle stiffness and the increased recruitment of motor units [69]. However, according to available evidence, performing RE on a vibrating platform has no particular contribution to improving running economy compared to RE alone [70].

### 2.6. Effects of RE Program Interruption

Interruption of the RE training results in the reduction of strength endurance, maximum strength, and power [71]. Energy expenditure levels during running are maintained even after a 2-week period without training [72]. A recent study investigated the effects of an 8-week explosive RE program, followed by a 4-week interruption (only endurance training was conditioned) of training, on running economy, V̇O_2_max, maximum strength, CMJ, and 3000 m running performance [71]. The 8-week program elicited significant improvements in running economy (+5.7%), which were retained after the 4-week period without RE training. Importantly, 3000 m run times were reduced after the first period (−2.4%), with a possible further reduction after 4 weeks without RE training (−4.4%). While this research was conducted on a very small sample and should be interpreted with caution, the results indicate that the incorporation of RE should be emphasized in the pre-competition phase, with a possible detraining or cessation of RE before the competition. Berryman et al. [71] also found that neuromuscular abilities were maintained after the interruption period. A meta-analysis found that a short-term interruption of resistance training reduces muscle strength, while the magnitude of the effect varies according to the type of training, age, and duration of the interruption [73]. However, this meta-analysis was not specific to RE in endurance runners. According to current findings, additional research is clearly needed in this area [71].

### 2.7. Acute Effects of RE and Interference with Running Training

It should also be pointed out that the acute effect of RE (both strength training and plyometric training) is associated with increased energy expenditure during running and thus reduced running economy, which could pose a problem for runners. Namely, RE could impact the quality of endurance training. Marcello et al. [74] examined the acute effects of a combination of strength and plyometric training on running economy in endurance runners. Elevated V̇O_2_ and post-workout energy expenditure indicated that strength and plyometric training reduced running economy at moderate running intensities; however, this negative effect disappeared in less than 24 h, which was also observed in a previous study [75]. Acutely, strength training leads to decreased muscle activation during running, leading to the reduced ability to produce force [76]. Similarly, activation inhibition occurs with plyometric training. Paschalis et al. [77] analyzed the effect of eccentric exercise on indirect indicators of muscle damage (delayed onset of muscle soreness, range of motion, and isometric force) and running economy. The training consisted of 12 × 10 repetitions of maximal voluntary contractions by each leg. Post-exercise indirect muscle damage markers were significantly altered after 24 to 72 h (*p* < 0.05), without significant changes in running economy (*p* > 0.05). Later, the same research group reported that after 48 h, post-exercise muscle damage indicators were significantly changed, while running economy (assessed at 70% VO_2_max) remained unchanged [78]. Burt et al. [79] measured the impact of exercise-induced muscle damage after performing 100 squats (10 sets of 10 repetitions at 80% of body mass) on the submaximal running performance of recreational athletes. They recorded a significant increase in V̇O_2,_ minute ventilation and blood lactate during submaximal running, as well as decreased maximum torque in hip extensors and lower vertical jump height 24–48 h post-training. Later, the same research group demonstrated that the repeated bout effect (the protective effect of an exercise session during subsequent bouts of muscle-damaging exercise [80]) also extends to running performance [81]. In sum, practitioners must keep the acute effects of RE in mind when designing RE programs for runners. The literature indicates that RE and the associated exercise-induced muscle damage may acutely interfere with running economy and that this effect is likely smaller after subsequent sessions of RE.

### 2.8. Additional Considerations

Strength, endurance, and energy expenditure during activity also affect triathlon performance [55]. In triathlons, each discipline has a negative impact on the amount of energy available for the next discipline [82]. One study [55] found increased cycling economy after endurance training; however, running economy was only improved with an addition of heavy weight RE. Concurrent resistance training and endurance training were presumed to increase musculoskeletal stiffness and to promote a greater recruitment of motor units, increasing the rate of force development and maximum strength [55], without changes in body mass. Similar findings were provided by other studies; for instance, Millet et al. [83] found that heavy weight training improved maximal strength and RE, but did not affect V̇O_2_.

In the current literature, there is a clear lack of studies investigating specific running populations, such as trail runners and ultra-endurance runners. During later stages of ultra-endurance events and during uphill running, the energy cost of running is increased [84]. Future studies could investigate if RE can ameliorate this decrease. Recent research indicates that muscle strength and power tests may be sensitive to the running-related workload [85]. In addition to classic RE, uphill running training may also improve running economy [86]. Although the method of running uphill is often used to improve running speed and, consequently, running performance, there is a lack of research on this topic, especially in terms of physiological adaptations [87]. One study compared the effects of high-intensity uphill and ground-level running intervals on V̇O_2_, blood lactate concentration, and muscle strength, and while there was an improvement in some outcomes (V̇O_2_, blood lactate concentration, and % V̇O_2_max) after both training regimens, the changes were very small [87]. Further research is needed to assess the effects of uphill running on running economy. As it currently stands, it seems that this method is inferior for improving running economy in comparison to RE.

## 3. Resistance Exercise for Modifying Running Technique

Running technique is an important aspect for movement economy and injury prevention. Several biomechanical factors were raised in the literature that may affect running economy. These factors can be categorized as spatiotemporal (parameters relating to changes in the phase of the gait cycle, such as ground contact time, stride length, etc.), lower limb kinematics (lower limb joint angles), kinetics (ground reaction forces), and neuromuscular factors (muscle activation and coactivation). Regarding the spatiotemporal factors, the literature reports that self-selected stride length and frequency is optimal or very near to being optimal for maximizing economy [88,89]. On the other hand, some of the literature reports that optimal stride length could be 3% shorter than preferred stride length for improving running economy [90,91]. Although there seems to be some merit in attempts to optimize stride frequency, such approaches are questionable for runners who have likely achieved optimal stride frequency through years of training. Stride length and stride frequency may also affect how far ahead of the body the foot strikes the ground. In running techniques with longer stride length and lower stride frequency, the foot strikes the ground far ahead of the body, with the first ground contact through the heels. Running on the heels is related to a higher breaking peak force [92] and could present an injury risk factor. On the other hand, a higher stride frequency decreases the length between the initial foot strike and center of mass (CoM), thus reducing peak braking and propulsive force [93]. Moreover, studies reported that a rear foot strike imposed higher biomechanical loads on overall ground impact and knee and patellofemoral joints, whereas a forefoot strike imposed higher biomechanical loads on the ankle joint and Achilles tendon [94]. When designing RE programs, running technique should be taken into account in order to implement exercises that target specific muscles and tissues to prepare them for running loads. Similar to stride frequency and stride length, vertical oscillation can be altered. Acute intervention studies have shown that increasing vertical oscillation leads to an increase in V̇O_2_ [95,96]. It is important to mention that rearfoot striking is more prevalent with increased distance within a running session/race and occurs more commonly in less experienced runners [97,98].

Furthermore, one of the reasons for the occurrence of overuse injuries is suboptimal running technique, resulting in poor lower limb kinematics [29,33], which results in the uneven distribution of forces between body segments. It is suggested that one of the contributing factors to the development of running-related injuries may be the suboptimal lower limb kinematics, especially the inability to control the motion of the lower extremity segments in the frontal and transverse planes. Examples from the literature include excessive hip adduction and internal rotation, knee abduction, tibial internal rotation, and foot pronation [99]. Moreover, a poor dynamic alignment pattern of hip adduction and internal rotation, knee abduction, tibial internal rotation, and foot pronation during running may lead to injury [99,100]. Biomechanical studies report that hip abductors and external rotators play an important role in controlling the position of the entire lower extremity in the frontal and transverse planes in the single-leg stance, suggesting that strengthening those muscles may benefit running technique and reduce injury risk. This was supported by Bellchamber and Van Den Bogert [101], reporting that the energy flow during walking is oriented from proximal to distal, suggesting that the kinematics of proximal joints affect the kinematics of distal joints, thus highlighting the importance of pelvis and hip musculature for running and walking. Therefore, it is possible that increased hip strength may affect the lower extremity biomechanics in the frontal and transverse planes and therefore improve the dynamic alignment of lower extremity joints, thus improving running technique. However, the literature on the effects of RE on running biomechanics is relatively scarce. The synthesis of the relevant studies is provided below.

Feltner et al. reported that an 8-week intervention consisting of exercise that directly strengthened the ankle and foot musculature (isokinetic eversion and inversion training) significantly reduced the inversion/eversion angle at heel strike and eversion range of motion [102]. However, this was not true for the functional training consisting of exercises such as step-ups, balance board exercises, and one- and two-leg jumps. Furthermore, Snyder et al. reported that a 6-week hip-strengthening program reduced foot eversion range of motion, hip internal range of motion, ankle inversion moment, and knee abduction moment, while there were no changes in pelvic range of motion in the frontal plane [103]. Together, these findings demonstrate that strength training oriented towards strengthening the hip and ankle/foot musculature reduces eversion range of motion, while strengthening the hip musculature also reduces hip internal range of motion and knee abduction when running. This indicates that strength training may influence the lower extremity joint loading, thus improving running technique.

Moffit et al. [104] found that maximal strength in squatting is associated with lower internal rotation of the knee during running. Contrary to those results, Willy and Davis reported that strength training intervention that improved hip abduction strength, hip external rotation strength, and single-leg squat mechanics did not alter running technique [105]. Similar results were also reported by Snyder et al. [103], indicating that running technique is difficult to change by strength training alone. On the other hand, the literature reports that running technique could be improved with neuromuscular training with feedback regarding critical technique deficits, such as, for example, hip adduction, hip internal rotation, and contralateral pelvis drop [106]. Moreover, feedback interventions are promising for reducing ground reaction forces during running [107,108].

In conclusion, RE for lower extremities may be beneficial for improving joint kinematics during running, but the impact on overall running technique is questionable, and more research is needed. Nevertheless, the literature does not report negative effects of RE on running biomechanics, suggesting that for improving running technique, neuromuscular training with feedback regarding technique deficits could be a favorable option.

## 4. Resistance Exercise for Reducing the Risk of Running-Related Injuries

Running-related injuries remain a major problem for runners [109], and the best approach to prevention is still largely equivocal [37,39]. The most prevalent running-related injuries are medial tibial stress syndrome, Achilles tendinopathy, plantar fasciitis, patellar tendinopathy, iliotibial band syndrome, stress fractures of the tibia, and patellofemoral pain syndrome [28,110]. Very recently, a study conducted on more than 4000 runners reported virtually no benefit of a comprehensive prevention program on injury risk [111]. The implemented program included various pieces of advice pertaining to load progression and management, footwear, subjective aspects, and running-specific exercises. However, RE was not included. Some of the running injuries (e.g., ITBS and PFPS) are characterized by altered running mechanics and weakness of certain muscle groups. The purpose of this section is to summarize the available evidence of the effectiveness of RE for reducing the risk of running-related injuries. According to Vincent et al. [112], more than 70% of running injuries are of the overuse nature, but inadequate running techniques and poor neuromuscular control are also key mechanisms for the occurrence of injuries. In trail runners, running experience, neglecting warm-ups, having no specialized running plan, training on asphalt, and double training sessions per day contribute significantly to injury risk [113].

### 4.1. Studies Investigating Running-Related Injuries in General

In a recent study [114], track and field runners reduced the incidence of injuries, especially medial tibial stress syndrome (*p* = 0.012), after 6 weeks of neuromuscular training, which included jumping, landing, strength, endurance, agility, and trunk training (*p* = 0.044), and they also improved their physical fitness. However, this was a very small study (*n* = 22), and the generalization of the effectiveness of such an exercise program is limited. A study of 1538 soldiers found that a 12-week stretching program during warm-up work did not reduce the risk of injury to soldiers; however, they found that the soldiers’ physical fitness itself was a statistically significant predictor of risk of lower limb injury [115]. In another cohort of soldiers, performing RE at least three times a week was associated with a 54% lower risk of running-related injuries compared to performing no or less than one unit of RE per week (*p* < 0.001) [116]. A 12-month combined prevention training exercise with a concomitant increase in physical exertion in 1020 soldiers did not result in a statistically significant reduction in the incidence of injuries (*p* = 0.162) [117]. A 12-week self-administered muscle strengthening program (included muscles: quadriceps, abductors, and trunk muscles), did not reduce the incidence of marathon injuries and the associated early end of the race (*p* = 0.90) [118].

Strengthening the ankle and foot muscles reduced the incidence of running-related injuries [119]. This one-year intervention differed from other interventions, as it focused primarily on the ankle–foot complex. The intervention strengthened the muscles of the ankle and foot, which improved cushioning upon contact with the ground, reduced the cumulative load, improved the movement in the ankle, and thus reduced the occurrence of running injuries. The intervention improved tolerance for the load, as the author found a correlation between foot strength and the time to injury. Lastly, the incidence of running injuries was 2.42 times higher in the control group, which did not undergo the intervention. These findings support the previously proposed protocol to strengthen the foot muscles to increase the ability to cushion forces upon contact with the ground [120]. The foot placement index was found to correlate with the longer period to injury occurrence. An 8-week foot and ankle muscle strengthening program increased the cross-section of the abductor hallucis and flexor digitorum brevis muscles in 31 healthy runners, but there were no changes in running biomechanics and toe muscle strength [121].

Peterson et al. [122] found in a meta-analysis that running injuries may be associated with reduced knee extensor strength (*p* = 0.03) and lower hip adduction velocity during running (*p* = 0.04). Running injuries may also be associated with an imbalance between stronger hip abductors and weaker hip adductors [123]. A study conducted on high school cross-country runners found an association between the decreased strength of hip abductors (*p* = 0.046), knee flexors (*p* = 0.046), and knee extensors (*p* = 0.038), and a higher incidence of anterior knee pain [124]. Another study found an association between running injury rate and asymmetry of hip abductor strength in male student runners, while in female runners, the injuries were associated with decreased unilateral hip abductor strength and bilaterally decreased hip external rotator strength [125].

### 4.2. Studies Assessing Specific Running-Related Injuries

According to Mucha et al. [126], decreased hip flexor strength in endurance runners is associated with the risk of ITBS. A study of 24 endurance runners with ITBS found that the strength of the hip abductors of the injured leg was lower than the strength of the hip abductors of the intact leg as well as the strength of the hip abductors of runners in the control group. In the second part of the study, a 6-week rehabilitation was performed, mainly aimed at strengthening the gluteus medius muscle, which increased the hip abduction strength in runners (women: +34.9% and men: +51.4%), as well as eliminated pain, thereby allowing runners to return to running without a recurrence of symptoms [127]. A recent pilot study [128] found that an 8-week strength program in people with ITBS, including hip-strengthening exercises with the gradual addition of complexity, reduced pain, increased muscle strength, and improved movement function compared to stretching and isolated hip exercises. The correct running technique is also of key importance in ITBS, as the kinetics and kinematics of the hip, knee, and ankle differ between runners with and without ITBS [129].

An important factor in the development of PFPS is the weakness of the hip joint muscles, especially the hip abductors [130]. With an intervention that included a 3-week hip muscle strengthening program, hip abductor strength improved by 32.69%, which is comparable to another previous study [131]. Nevertheless, the study did not show a difference in running mechanics between the groups, so according to the researcher’s findings, the weakness of the hip abductors is not associated with altered running mechanics. A 14-week hip strengthening program on two subjects with increased hip adduction, internal hip rotation, and knee valgus, as well as decreased strength of the hip abductors, hip extensors, and external rotators of the hip, decreased knee pain and improved the mechanics of the lower limb when stepping down [131].

Although more typical for team sports and related to sprinting [132,133], hamstring injuries also occur in distance runners [28]. The risk of hamstring injury can be reduced by flexibility training as well as strength training, as Wan et al. [134] found that mobility training statistically significantly lengthened the semimembranosus and long head of the biceps femoris (*p* ≤ 0.026) and reduced the maximum load on the hamstrings, while strength training increased the length and reduced maximum load on all hamstring muscles. In their literature review, Raya-Gonzalez et al. [135] found that, among many other approaches, the reduction of hamstring muscle injuries is thought to be largely influenced by eccentric and balance training. In two case reports performed on two different runners with an inflamed hamstring tendon, the eccentric exercise protocol for the posterior thigh muscle, stabilization exercise for the lumbo-pelvic area, and dry needling trigger points reduced pain and tenderness as well as restored full lower limb function and running without the onset of symptoms [136].

Groin pain is a very common problem in professional athletes [137]. Due to the presence of specific anatomical structures in the groin area, the diagnosis of pain is often difficult, and the terminology appears to be inconsistent [138]. In the literature, sports hernias and chronic groin pain can also be found as synonyms for groin pain [137,138]. The presence of other pathologies is usually also reported in athletes with chronic groin pain [137]. Although groin pain is more common in athletes who perform repetitive hip movements (e. g. kicking the ball) and rapidly change the direction of running (team sports such as football and basketball), groin pain sometimes occurs in runners, as well [138]. Moran and Rogowski [138] performed exercises for hip stability and strengthening the pelvic and lumbar region on a female runner diagnosed with groin pain and labral lacerations. The intervention improved running cadence and stride, knee control, as a smaller knee valgus was observed, and decreased pelvic drop, enabling the runner to successfully return to competitions. After reviewing the literature, we found that very little literature has examined groin pain in runners and its association to running biomechanics [138]. Future research to examine the effects of RE interventions to reduce the risk of groin pain or prevent its reoccurrence is needed.

In female endurance runners, stress fractures are highly prevalent and may be associated with menstrual disorders. Hutson et al. [139] found that amenorrheic runners had a 2.25 higher incidence rate of stress fractures compared to eumenorrheic runners. In the same study, plyometric training did not seem to reduce the injury rate [139].

In summary, some studies indicate a potentially beneficial effect of RE on running-related injury risk. However, several studies have used small sample sizes and many have been performed on military personnel; thus, the generalization of the results is questionable. Previous literature reviews on the topic also reported inconclusive results [39,140] and stressed that more research is needed.

## 5. Conclusions

The aim of this narrative review is to summarize the available evidence on the effectiveness of RE for improving running economy, modifying running technique, and reducing the risk of running-related injuries. The evidence consistently shows that lower limb RE is effective for improving running economy. A combination of strength training and plyometric drills is recommended to improve RE. Isometric training is also emerging as a possible alternative and is recommend especially when the overall training load is already high. Lower limb RE may be beneficial for improving joint kinematics during running; however, biofeedback training is probably more effective for altering overall running biomechanics. RE may help reduce running-related injury risk, although the evidence is scarce. In this regard, we recommend that all major lower limb muscle groups be targeted if no specific deficits are present.

## Data Availability

Not applicable.
